# Histopathologic spectrum of childhood tumours in a Tertiary Hospital: a ten-year review

**DOI:** 10.4314/ahs.v21i1.9

**Published:** 2021-03

**Authors:** Said M Amin, Vincent E Nwatah, Emmanuel A Ameh, Abdurasaq R Oyesegun, Adewumi B Oyesakin

**Affiliations:** 1 Department of Histopathology, National Hospital Abuja, Nigeria; 2 Department of Paediatrics, National Hospital Abuja, Nigeria; 3 Division of Paediatric Surgery, Department of Surgery, National Hospital, Abuja, Nigeria; 4 Department of Oncology, National Hospital Abuja, Nigeria

**Keywords:** Childhood tumours, spectrum, haemangioma, lymphoma

## Abstract

**Background:**

There has been a growing public health burden of childhood tumours in low and middle income countries (LMICs) as the trend in epidemiological transition continues to vary.

**Objective:**

The objective of this report is to determine the spectrum of childhood tumours at a tertiary hospital in Nigeria.

**Methods:**

A retrospective review of the histopathology register over the period January 2006 to December 2015.

**Results:**

The total paediatric tumour cases was 248, including 143 (57.7%) females and 105 (42.3%) males, aged 0 – 12 years (mean 6.1 years ± 3.97 SD). The age group 2 – 5 year cohort had the highest prevalence of tumour. The predominant tumour based on tissue of origin was epithelial neoplasms 88 (35.5%), vascular neoplasms 56 (22.6%), neural neoplasm 42 (16.9%), mesenchymal neoplasm 37 (14.9%), germ cell neoplasm 13 (5.2%) and haematopoietic neoplasms 12 (4.8%). Majority of the tumours were benign, 148 (59.7%) and malignant 100 (40.3%). The most predominant benign tumour was haemangioma 33 (13.3%) and predominant malignant tumour was lymphoma 22 (8.9%).

**Conclusion:**

Benign tumours remain the commonest neoplasm of children in this hospital-based data. Development and implementation of a tumour registry would provide a more comprehensive information.

## Background

There has been an increasing trend in cancer rates in low and middle income countries (LMICs)^1^ due to the different forms of transitioning occurring in these countries. It is becoming an important cause of childhood morbidity and mortality in many LMICs. These LMICs are plagued with dearth of epidemiological data as well as low survival rates due to several factors.

Childhood cancers constitute about 0.5 to 2% of the total cancers in developed countries^2^, ^3^, ^4^. A true proportion of childhood cancers in developing countries may not be easily obtained due to under-reporting or lack of relevant data. Marked variation exist in the spectrum of childhood tumours seen across countries and geographical regions. This spectrum also differ from that found in adult population and even within countries^5^, ^6^. The general outcome for childhood tumours is poor especially in LMICs that are faced with huge challenges affecting diagnosis and treatment.^7^ Others^8^,^9^ have noted inadequate diagnostic skills and poor record keeping as mitigating factors prevalent in LMICs. The need for cancer registries have been advocated^3^, ^10^, ^11^. The aim of this report is to highlight the histopathologic spectrum of childhood tumours at a tertiary hospital in Nigeria, to enhance the determination of the epidemiology of these tumours in LMICs.

## Material and methods

### Setting

National Hospital Abuja is located in the Central business district of Federal Capital Territory (FCT) Abuja. As a referral centre it provides tertiary level health care for patients within Abuja as well as neighbouring cities and states. Abuja is the federal capital of Nigeria and occupies an area of about 8000sq.km. It is bounded by various states (North-Kaduna state, West-Niger, East-Plateau, South- East- Nasarawa and South-West- Kogi).

### Method

This was a retrospective review of histologic samples of children aged 0 – 12 years, with a histologic diagnosis of a childhood tumour from January 2006 – December 2015, at the National Hospital, Abuja. Information was extracted from the histopathology register and histopathology records at the pathology department of the hospital. Histology was performed using routine Hematoxylin and Eosin staining, and immunohistochemistry where necessary.

Ethical approval was obtained from the ethics and institutional review committee of the hospital.

### Data analysis

Data were entered into and analysed with statistical package for social science version 21 (SPSS Inc). Quantitative continuous variables like age were summarized using mean, standard deviation while categorical variables like sex were summarised using percentage. Results were presented in table and charts. Pearson's chi-square (χ^2^) was used to test the association between two categorical variables and a p-value of < 0.05 was considered statistically significant.

## Result

### Demographics

A total of 2967 solid tumours were diagnosed during the study period of which 248 (12%) occurred in children aged 0–12yrs.

There were 105 males and 143 females aged 0 – 12 years (mean 6.1 years ± 3.97). The age group 2 – 5 year cohort had the highest prevalence of tumour (Table 1).

**Table 1:** Age group, sex and neoplasm type distribution

Age group	Neoplasm type				Total N (%)	χ^2^	df	P-value
	Benign N (%)		Malignant N (%)					
	Male	Female	Male	Female				
**0 – 28dys**	0 (0)	8 (3.2)	2 (0.8)	0 (0)	10(4.0)	6.294	4	0.17
**29 – 1yr**	3 (1.2)	15 (6.0)	5 (2.0)	6 (2.4)	29(11.7)			
**2 – 5yrs**	21 (8.5)	23 (9.3)	18 (7.2)	17 (6.9)	79(31.9)			
**6 – 9yrs**	20 (8.1)	23 (9.3)	14 (5.6)	12 (4.8)	69(27.8)			
**10 – 12yrs**	13 (5.2)	32 (12.9)	9 (3.6)	7 (2.8)	61(24.6)			
**Total**	57 (23)	101 (40.7)	48 (19.4)	42 (16.9)	248(100)			

One hundred and fifty eight (63.7 %) tumours were benign and 90 (36.3%) malignant. Malignant histology was more prevalent in the 2 – 5 years age group 35 (14.1%) and benign histology in 10 – 12 years age group 45 (18.1%). There was no statistical correlation between the tumour type and age, p value 0.17.

**Figure 1:**
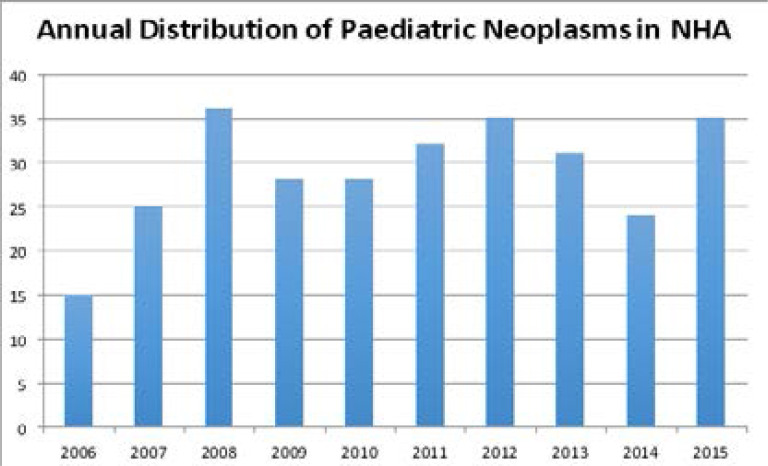
Distribution of paediatric neoplasms over the years

Figure 1 shows the yearly distribution of the tumours with 2008 having the highest prevalence. The yearly trend for childhood tumours showed peaked prevalence for 2008, 2012 and 2015.

### Tumour-type

Malignant histology was more prevalent in the 2 – 5 years age group 37(14.9%) and benignistology in 10 – 12 years age group 43(17.3%).

**Benign Tumours (Table 2):** the benign tumours were; hemangioma 33 (13.3%), papilloma 28 (12.3%), lipoma 17 (6.9%), neurofibroma 14 (5.6%) and others 30 (12.1%).

**Table 2:** Age distribution of benign neoplasms

Histological diagnosis	Age groups					

	0 – 28dys	29dys – 1yr	2 – 5yrs	6 – 9yrs	10 – 12yrs	Total N (%)
**Hemangioma**	0	5	3	13	12	33(13.3)
**Papilloma**	0	2	14	7	5	28(12.3)
**Lipoma**	0	2	9	4	2	17(6.9)
**Neurofibroma**	0	0	5	5	4	14(5.6)
**Fibroma**	2	1	0	5	5	13(5.2)
**Teratoma**	1	3	4	1	0	9(4.0)
**Cystic hygroma**	1	3	1	1	2	8(3.2)
**Adenoma**	1	0	2	2	1	6(2.4)
**Others** §	3	2	6	5	14	30(12.1)
**Total**	8	18	44	43	45	158(63.7)

§ Others include tumours with fewer frequencies less than 6 like; Adenoma (3), Enchondroma (1), Harmatoma (2), Histiocytoma (3), Fibroadenoma (5), Fibromyolipoma (1), Leiomyoma (1), Lymphangioma (3), Meningioma (2), Messenchynoma (1), Ossifying Fibroma (2), Osteochondroma (2), Papillomatosis (3), Xanthogranuloma (1).

**Malignant Tumours (Table 3):** the malignant tumours were; lymphomas 22 (8.9%), nephroblastoma 13 (5.2%), teratoma 10 (4.0%), carcinoma 10 (4.0%), retinoblastoma 7 (2.8%) and others 29 (11.7%).

**Table 3:** Age distribution of malignant neoplasm

Histological diagnosis	Age group					

	0 – 28dys	29dys – 1yr	2 – 5yrs	6 – 9yrs	10 – 12yrs	Total N (%)
**Lymphoma**	1	2	6	7	6	22(8.9)
**Nephroblastoma**	0	2	7	3	1	13(5.2)
**Carcinoma**	0	0	6	2	2	10(4.0)
**Retinoblastoma**	0	2	4	1	0	7(2.8)
**Neuroblastoma**	0	1	4	0	0	5(2.0)
**Medulloblastoma**	0	1	1	1	1	4(1.6)
**Others** §	1	3	7	12	6	29(11.7)
**Total**	2	11	35	26	16	90(36.3)

§ Others include tumours with fewer frequencies less than 4 like; Adenocarcinoma (1), Angiosarcoma (1), Astrocytoma (3), Dermatofibrosarcoma (2), Dysgerminoma (1), Ependymoma (1), Fibrosarcoma (2), Gasrointestinal stromal tumour (1), Hepatic haemangioendothelioma (1), Hepatoblastoma (3), Medulloblastoma (2), Melanocarcinoma (1), Melanoma (1), Neuroectodermal tumour (1), Osteosarcoma (2), Pinealoblastoma (1), Rhabdomyosarcoma (3), Sacrococcygeal teratoma (2).

The most prevalent tumours based on age groups were: fibroma 2 (0.8%) for neonatal period; haemangioma 5 (2.0%) for infancy, papilloma 14 (5.6%) for 2 – 5 years cohort; hemangioma 13 (5.2%) 6 – 9 years cohort; hemangioma 12 (4.8%) for 10 – 12 years cohort (table 2 and 3).

The tissue of origin (Table 4) were epithelial neoplasms 88 (35.5%), vascular neoplasms 56 (22.6%), Neural neoplasm 41 (16.5%), mesenchymal neoplasm 38 (15.3%) and germ cell neoplasm 13 (5.2%).

**Table 4:** Age group distribution based on tissue of origin

	Age groups N (%)					

	0–28dys	29dys–1yr	2–5yrs	6–9yrs	10–12yrs	Total
**Tissue of Origin**						
**Epithelial**	3(1.2)	9(3.6)	37(14.9)	21(8.5)	18(7.3)	88 (35.5)
**Neural**	0(0)	4(1.6)	15(6.0)	13(5.2)	9(3.6)	41 (16.5)
**Vascular**	1(0.4)	10(4.0)	15(6.0)	15(6.0)	15(6.0)	56 (22.6)
**Hematopoietic**	1(0.4)	1(0.4)	1(0.4)	5(2.0)	4(1.6)	12 (4.8)
**Mesenchymal**	3(1.2)	1(0.4)	6(2.4)	14(5.6)	14(5.6)	38 (15.3)
**Germ cell**	2(0.8)	4(1.6)	5(2.0)	1(0.4)	1(0.4)	13 (5.2)
**Total**	10(4.0)	29(11.7)	79(31.9)	69(27.8)	61(24.6)	248 (100)

## Discussion

The hospital prevalence of childhood tumours of 12% of all tumours and annual case rates are similar to other reports from Nigeria^8^, ^9^, ^12^, ^13^. Report from other countries have also showed varying higher rates^14^, ^15^. The variations noted between these studies could be attributed to differences in study scope as some studied only solid tumours or malignant tumours. The current study did not include leukemia which may have contributed to the relative lower prevalence of childhood tumour in comparison to other countries. Also the study was hospital based and study centre was situated at the nation's capital, not every patient reached the hospital and some patients may have died before getting to the hospital. Early childhood, 2 – 5 years cohort were found to have the highest prevalence of malignant tumours while 10 -12yrs had the highest prevalence of benign tumours. Some authors^5^, ^14^ also reported similar findings in their study though they concentrated on only malignant solid tumours. Conversely the late childhood period 5 – 9 years was found to have higher risk for cancers by some authors.^15^, ^17^ The reason for more prevalent malignant tumour among the under-5 may not be very apparent in this study due to the study limitations such as lack of detailed demographics and clinical data. However genetic and several environmental factors have been linked as risk factors but for many cases of childhood malignancies the cause and risk factors remains unknown.

Haemangioma was the commonest benign tumour while lymphoma accounted for the commonest malignant tumour. Nigeria is within the malaria endemic sub-Saharan region. The high prevalence of lymphoma may be associated with the regional high prevalence of malaria. Similarly, other authors^14^, ^15^, ^17^ found lymphoma to be most prevalent malignant tumour in their studies. However, other studies have reported varying tumours like retinoblastoma, rhabdomyosarcomas and nephroblastoma as being most prevalent^8^, ^9^, ^16^. It may be difficult to ascertain the factors influencing these differences as ethnicity, geographical location and environmental exposures were not explored in these studies. Distribution of tumours across age groups varied as expected with harmatoma and haemangioma more common in the neonatal and infancy period respectively. The predominance of harmatoma and haemangioma in this age group reflects the embryogenesis of vascular tumours as well as the latency period for childhood malignant tumour to occur. One study from western Nigeria^8^ reported only retinoblastoma within the neonatal period.

As highlighted by Stefan et al^2^ diagnosis and notification of childhood cancer should be improved more especially in resource poor settings. Bridging the existing gap of insufficient or lack of record keeping by development of paediatric tumour registry will assist in determining the epidemiology and trend of this emerging public health issue especially in developing countries. The lack of cancer registry is a major limitation in LMICs. The study being a retrospective review of records not intended as registry limit the use of inferential analysis as many associating factors could neither be ascertained nor substantiated in this study. Such factors like geographical/ethnic demographics, pattern of presentation, diagnosis-intervention intervals and outcome variables would have been necessary to determine if appropriate registry was established. Also lack of clinical data in the review limits the application of the findings.

## Conclusion and recommendation

Benign tumours remain the commonest neoplasm of children in this hospital-based data. Development and implementation of a tumour registry would provide a more comprehensive picture.
